# Associations between prospective symptom changes and slow-wave activity in patients with Internet gaming disorder: A resting-state EEG study

**DOI:** 10.1097/MD.0000000000006178

**Published:** 2017-02-24

**Authors:** Yeon Jin Kim, Jun-Young Lee, Sohee Oh, Minkyung Park, Hee Yeon Jung, Bo Kyung Sohn, Sam-Wook Choi, Dai Jin Kim, Jung-Seok Choi

**Affiliations:** a Department of Psychiatry, SMG-SNU Boramae Medical Center; b Department of Psychiatry and Behavioral Science, Seoul National University College of Medicine; c Department of Biostatistics, SMG-SNU Boramae Medical Center; d Korea Institute on Behavioral Addictions, True Mind Mental Health Clinic, Seoul; e Korea Health Care and Information Research Institute, Namseoul University, Cheonan; f Department of Psychiatry, Seoul St. Mary's Hospital, The Catholic University of Korea College of Medicine, Seoul, Republic of Korea.

**Keywords:** Internet gaming disorder, resting-state EEG, slow-wave activity, treatment response

## Abstract

The identification of the predictive factors and biological markers associated with treatment-related changes in the symptoms of Internet gaming disorder (IGD) may provide a better understanding of the pathophysiology underlying this condition. Thus, the present study aimed to identify neurophysiological markers associated with symptom changes in IGD patients and to identify factors that may predict symptom improvements following outpatient treatment with pharmacotherapy. The present study included 20 IGD patients (mean age: 22.71 ± 5.47 years) and 29 healthy control subjects (mean age: 23.97 ± 4.36 years); all IGD patients completed a 6-month outpatient management program that included pharmacotherapy with selective serotonin reuptake inhibitors. Resting-state electroencephalography scans were acquired prior to and after treatment, and the primary treatment outcome was changes in scores on Young's Internet Addiction Test (IAT) from pre- to posttreatment. IGD patients showed increased resting-state electroencephalography activity in the delta and theta bands at baseline, but the increased delta band activity was normalized after 6 months of treatment and was significantly correlated with improvements in IGD symptoms. Additionally, higher absolute theta activity at baseline predicted a greater possibility of improvement in addiction symptoms following treatment, even after adjusting for the effects of depressive or anxiety symptoms. The present findings demonstrated that increased slow-wave activity represented a state neurophysiological marker in IGD patients and suggested that increased theta activity at baseline may be a favorable prognostic marker for this population.

## 1 Introduction

Internet gaming disorder (IGD) refers to compulsive and problematic use of Internet-based games that interferes with normal daily life.^[1]^ Previous research has shown that various psychiatric comorbidities are associated with IGD, including depressed mood,^[2]^ anxiety,^[3]^ attention deficit hyperactivity disorder,^[4]^ and obsessive–compulsive disorder.^[5]^ Electroenonecephalographic (EEG) studies of spontaneous brain activity during resting conditions with the eyes closed have been increasingly recognized as a useful method for the investigation of the neural correlates of cognition and behavior.^[6]^ Spontaneous brain activity is neural activation in the absence of an explicit task, such as sensory input or motor output, and, hence, is referred to as resting-state activity.^[7]^ This type of neural activity reflects the brain's ability to allocate resources and prepare for changes in the internal and external environments.^[8]^ Thus, the examination of resting-state EEG data as a measure of cognitive capabilities may enhance the current understanding of basic brain functions.

Several studies have used resting-state EEG to investigate the characteristics of patients with IGD. Absolute beta power is lower in these patients under resting-state conditions and is correlated with standard self-report measures of impulsivity.^[9]^ Furthermore, the resting-state EEG activity of IGD patients aids in the differentiation of this population from patients with alcohol use disorder (AUD) because IGD patients have lower absolute beta power than AUD patients and healthy controls. However, this marker is not related to the severity of IGD, which suggests that decreased beta power is a trait marker. In contrast, AUD patients exhibit higher absolute delta power than do patients with IGD and healthy controls.^[10]^ IGD patients also frequently exhibit comorbid depressive or anxiety symptoms; thus, it is plausible that the comorbidity of these conditions may influence resting-state brain activity. For example, Lee et al^[11]^ found that IGD patients with depression show increased absolute power in the delta band, increased relative theta, and decreased relative alpha powers in all regions, especially fronto-central regions, which are known to be closely related to depression. Therefore, it is necessary to diagnose the individual comorbid psychiatric symptoms of IGD patients to accurately characterize the neurobiological characteristics associated with this disorder.

The identification of biological markers associated with treatment-related changes in IGD symptoms can provide a better understanding of the pathophysiology underlying IGD. Because IGD is associated with a poor quality of life and lowered psychological well-being,^[12]^ the early identification of treatment responders and the implementation of early interventions involving individualized treatment approaches would likely have significant clinical importance. Abstaining AUD patients show an increase in slow-wave brain activity after 6 months of treatment compared with baseline measurements, which indicates a normalization of brain function.^[12]^ Recently, Wang et al^[13]^ suggested that abnormal resting-state electrical activity, including increased power in the theta band, in the brains of patients who use illicit opiates may be reduced by methadone treatment. Moreover, an event-related potential study found that the P300 amplitude reliably predicts treatment completion in substance-dependent populations;^[14]^ however, this peak was not recorded during the resting state.

To date, no studies have investigated the neurophysiological factors associated with treatment responses in patients with IGD. Thus, the present study aimed to determine the neurophysiological markers associated with symptom changes in IGD patients with comorbid psychiatric issues and to detect markers that will predict symptom improvements following outpatient management with pharmacotherapy. Based on previous studies of patients with IGD and/or substance use disorders,^[11,13,14]^ it was hypothesized that IGD patients with comorbid symptoms would exhibit increased activities in the delta and theta bands on the fronto-central regions and also associations between slow-wave activity in the delta and theta bands and improvements in addiction symptoms following 6 months of outpatient management.

## 2 Methods and materials

### 2.1 Study participants

The present prospective longitudinal study included 49 male participants, 20 of whom were IGD patients (mean age: 22.71 ± 5.47 years) and 29 of whom were healthy control subjects (mean age: 23.97 ± 4.36 years). All patients had sought treatment at outpatient clinic of SMG-SNU Boramae Medical Center in Seoul, South Korea due to excessive participation in Internet gaming, and IGD was diagnosed by an experienced psychiatrist based on the criteria of the Diagnostic and Statistical Manual of Mental Disorders, Fifth Edition (DSM-5). Additionally, Young's Internet Addiction Test (IAT) was administered to assess the severity of IGD symptomatology.^[15]^ The IAT has been extensively used in the research field of IGD,^[16]^ and well documented its psychometric properties in different population and languages.^[17–19]^ A recent validation study for Korean translation of the IAT showed outstanding internal consistency and high test–retest reliability.^[20]^ These items are rated on a 5-point scale ranging from 1 (very rarely) to 5 (very frequently); in the present study, IAT scores were calculated with the total score for all 20 items ranging from 20 to 100. The present study included only those patients with IAT scores ≥70 who spent more than 4 hours per day and 30 hours per week using Internet games to clarify the pathological changes associated with IGD and also to evaluate only patients with severe IGD rather than patients at a high risk of developing this disorder. Additionally, to assess lifetime psychiatric diagnoses, the Structured Clinical Interview for DSM-IV disorders was used.

Following the completion of the clinical assessments and a baseline EEG scan, the IGD patients completed a 6-month outpatient management program that included pharmacotherapy with selective serotonin reuptake inhibitors using the average following doses: escitalopram at 15.83 ± 9.17 mg, fluoxetine at 50.00 ± 9.17 mg, and paroxetine at 30.00 ± 14.14 mg. All IGD patients who completed the 6-month treatment program had comorbid depressive or anxiety symptoms at baseline and completed a follow-up EEG scan upon finishing. The primary treatment outcome of the present study was change in IAT score from pre- to posttreatment, and the Beck Depression Inventory (BDI)^[21]^ and Beck Anxiety Inventory (BAI)^[22]^ were administered at the pre- and posttreatment assessments of the IGD patients to evaluate changes in comorbid depressive and anxiety symptoms. Healthy control subjects were directly recruited from the local community, did not have a history of psychiatric disorders, and played Internet games less than 2 hours per day.

Participants were excluded from the present study if they had a history of significant head injury, seizure disorder, intellectual disability, psychotic disorder, or substance use disorder (other than one involving nicotine). Additionally, all participants were medication-naive at the time of the baseline assessment. The Korean version of the Wechsler Adult Intelligence Scale-III was administered to all subjects to estimate their IQ; only individuals with Wechsler Adult Intelligence Scale-III scores ≥80 were included in the present study. The Institutional Review Board of the SMG-SNU Boramae Medical Center approved the study protocol, and all subjects provided written informed consent prior to participation.

### 2.2 EEG recording

The detailed protocols for the EEG recording and data acquisition procedures have been described in previous reports published by our research group.^[9–11]^ Briefly, participants were engaged in a resting state, while seated in a darkened noiseless room that was connected to a recording room via a 1-way glass window. The EEG recording was conducted for 10 minutes under the following conditions: 4 minutes with eyes closed, 2 minutes with eyes open, and 4 minutes with eyes closed.

The EEG recordings and acquisitions were obtained using SynAmps2 with a 64-channel Quik-cap and the NeuroScan system (Scan 4.3, Compumedics Ltd; Abbotsford, Australia). A single channel with bipolar electrodes was attached to the mastoids as a reference, and a ground channel was placed between the FPz and Fz electrodes. The EEG signals were obtained at a sampling frequency of 500 Hz and band-pass filtered at 0.1 to 60 Hz using Scan 4.3 with an electrode impedance below 5 Kω. The recordings from the NeuroScan system were transferred to NeuroGuide software (NG 2.5.5, Applied Neuroscience, Inc, St. Petersburg) for spectral analysis in a 32-bit file format, and 19 of the 64 channels were driven by the following NeuroGuide montage set: FP1, F3, F7, Fz, FP2, F4, F8, T3, C3, Cz, T4, C4, T5, P3, O1, Pz, T6, P4, and O2.

Artifact removal was performed off-line using the artifact rejection toolbox in the NeuroGuide software. Additionally, all EEG recordings were visually analyzed to eliminate signal distortions, such as eye muscle movements. Samples were selected by visual inspection to obtain a minimum of 20 to 60 seconds of data during the eyes-closed resting state for the spectral analysis, and the accepted epochs of EEG data for both the absolute (actual spectral power; uV^2^) and relative (proportion of spectral power in the combined sum of frequency bands; %) powers were smoothed using fast Fourier transforms. The power was divided into 4 frequency bands and averaged with the NeuroGuide spectral analysis system as follows: delta (0.5–4 Hz), theta (4–8 Hz), alpha (8–12 Hz), and beta (12–30 Hz). In the analysis of the present study, we focused on the delta and theta bands of slow-wave. Additionally, based on previous studies, we analyzed EEG activity from 19 selected sites that were divided into 3 regions by averaging within each region: frontal (FP1, F3, F7, Fz, FP2, F4, and F8), central (T3, C3, Cz, T4, and C4), and posterior (T5, P3, O1, Pz, T6, P4, and O2).^[9–11]^

### 2.3 Statistical analysis

Prior to the formal analyses, an exploratory data analysis was conducted to identify and remove outliers to avoid the possibility of spurious results. Because repeated or multiple outcomes from the same subject are correlated, the present study utilized a generalized estimating equation,^[23]^ which is an extension of the generalized linear model for multivariate responses, to assess group effects on the absolute and relative powers in each delta and theta frequency band. The generalized estimating equation was employed to test group (IGD vs. healthy control), region, and the interaction of these factors in each frequency band because this model has previously been used to analyze EEG characteristics.^[24–26]^ Significant interactions between the groups in different regions were calculated using a group-by-region interaction because it reflects the group effects on the absolute and relative powers for each frequency band according to region. In the absence of an interaction effect, the group effects were also tested.

Independent-sample *t* tests were performed to compare the demographic and clinical variables of the 2 groups, whereas differences in clinical symptoms and EEG data between the pre- and posttreatment assessments were analyzed with a repeated measure analysis of variance after adjusting for BDI and BAI scores. Additionally, a multiple linear regression analysis was conducted to examine associations among the baseline EEG markers and changes in IAT scores (IAT score at pretreatment − IAT score at posttreatment) after adjusting for BDI and BAI scores. A Pearson correlation analysis was performed to examine relationships among changes in EEG markers and changes in IAT scores after 6 months of treatment. All statistical analyses were performed with the SPSS Statistics package, version 20 (IBM Inc; NY) and R version 2.15.2 (http://www.r-project.org). *P* values <0.05 were considered to indicate statistical significance.

## 3 Results

### 3.1 Demographic and clinical/cognitive data

Table 1 summarizes the demographic and clinical characteristics of the participants. There were no significant differences between the IGD patients and healthy control subjects in terms of age, education, or IQ, but the IGD patients had higher BDI (*P* < 0.001) and BAI (*P* < 0.001) scores than the controls. After 6 months of treatment, the IGD patients exhibited a significant decrease in IAT scores (*P* < 0.001) but not in BDI and BAI scores.

**Table 1:**
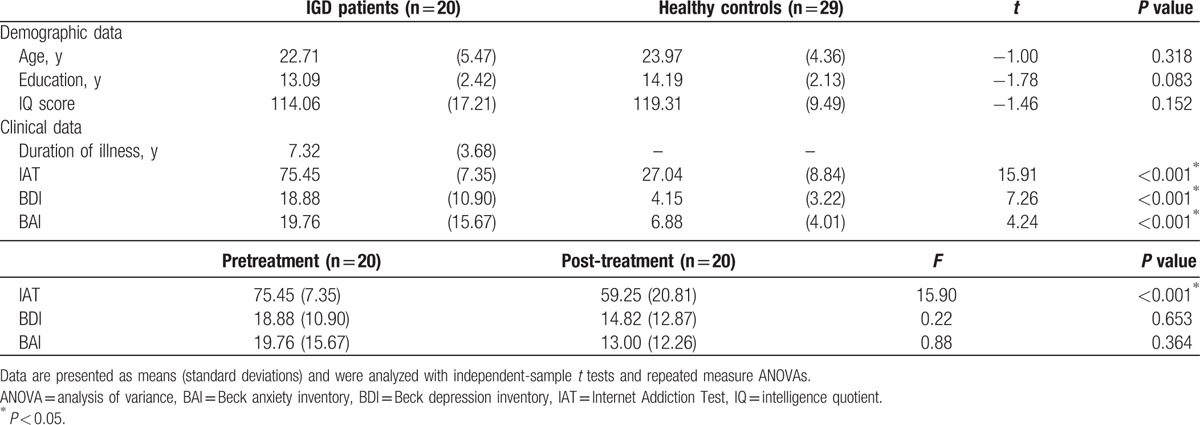
Demographic and clinical characteristics of study subjects.

### 3.2 Quantitative changes in EEG data following treatment

**Figure 1:**
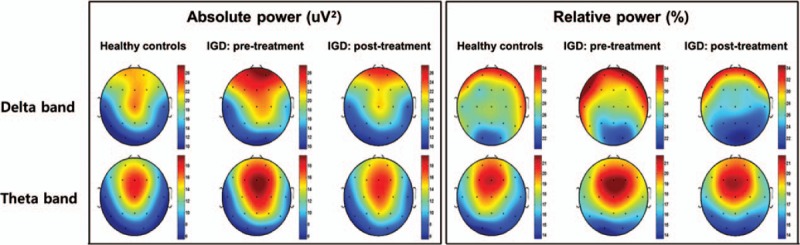
Topographical maps of the absolute and relative powers in patients with Internet gaming disorder (IGD) before and after the 6-month outpatient treatment and the healthy control group at baseline. Scales show uV^2^ for absolute power and % for relative power. Red represents higher values, and blue represents lower values.

**Figure 2:**
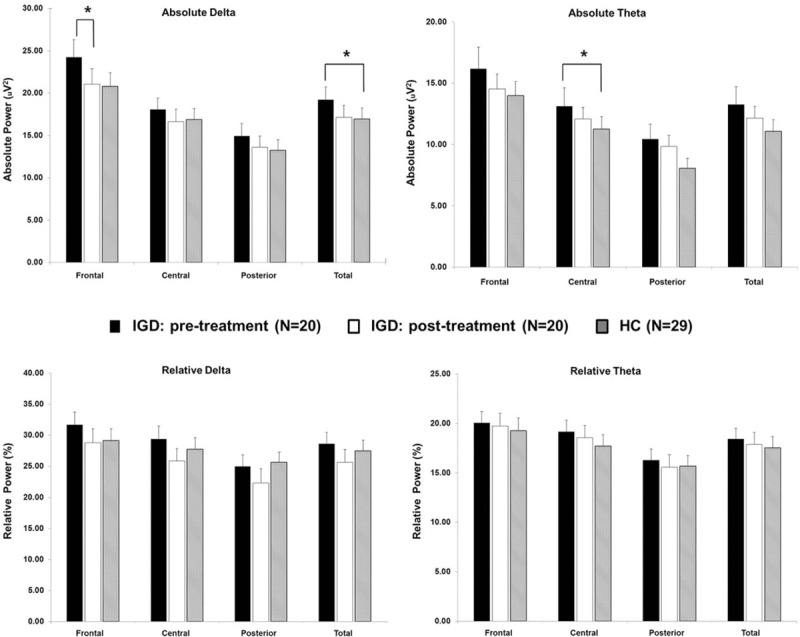
Absolute and relative powers in each band under the resting-state eyes-closed condition. Horizontal bars represent standard deviations. ∗ *P* < 0.05.

Figure 1 illustrates the scalp topographies of patients in the IGD group before and after treatment in terms of the absolute and relative powers in each band. Compared with the healthy control group, the IGD group had increased absolute powers in the delta band of the total brain (estimate = 4.96, *t* = 2.05, *P* = 0.046) and in the theta band of the central brain region (estimate = 5.15, *t* = 2.41, *P* = 0.021; Fig. 2). There were no group-by-region interaction effects for the absolute or relative powers in any of the other bands, and the main group effects were not significant in any of the regions.

Following 6 months of treatment, the absolute power in the delta band of the frontal region of the IGD group exhibited a significant reduction compared with baseline (*z* = 2.03, *P* = 0.043), and the extent of this decrease was significantly correlated with change in IAT score (*r* = 0.57, *P* = 0.011). In contrast, there were no significant absolute or relative changes in any of the other bands following treatment nor were there significant differences in the absolute or relative powers in any region between the IGD group at the posttreatment assessment and the healthy control group. Further, the reduction of the frontal delta activity was not correlated with changes in depressive (*r* = −0.08, *P* = 0.778) or anxiety symptoms (*r* = −0.02, *P* = 0.935) following treatment in the IGD group.

### 3.3 Quantitative EEG components predictive of improvements in IGD symptoms

**Figure 3:**
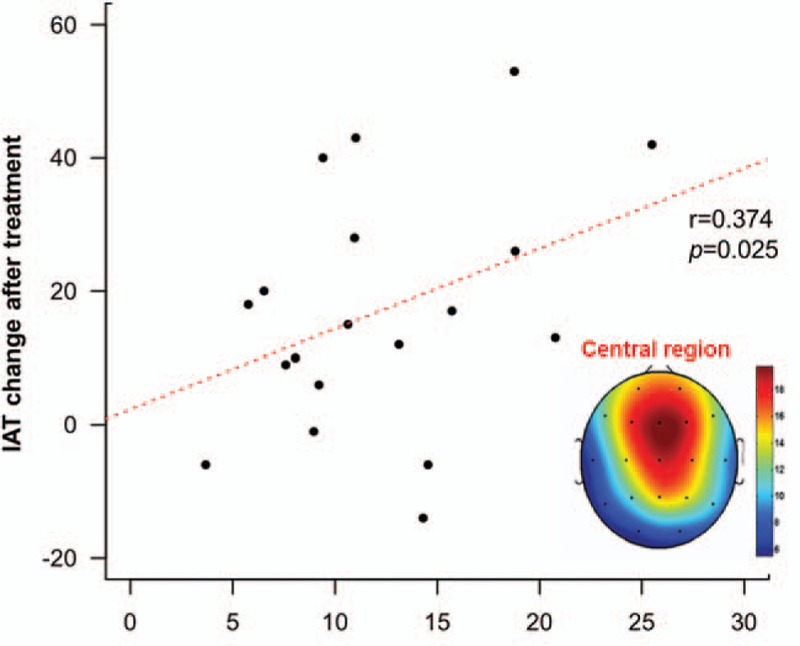
Relationship between absolute power in the theta band of the central region at the pretreatment assessment and change in IAT score after 6 months of treatment in patients with IGD. Higher absolute theta activity in the central region at baseline predicted a greater possibility of improvements in addiction symptoms after treatment in IGD patients with comorbid problems. IAT = Young Internet Addiction Test, IGD = Internet gaming disorder.

A multiple regression analysis revealed that change in IAT score was significantly predicted by absolute power in the theta band of the central brain region at baseline (estimate = 3.10, *t* = 2.53, *P* = 0.025; Fig. 3). In other words, higher absolute theta activity in the central region at baseline predicted a greater possibility of improved addiction symptoms in IGD patients with comorbid issues following treatment, even though the effects of depressive or anxiety symptoms at baseline were controlled.

## 4 Discussion

The present study was the first to investigate the neurophysiological changes associated with symptom improvement in IGD patients. IGD patients with comorbid depressive or anxiety symptoms showed increased resting-state EEG activity in the delta and theta bands at baseline, but this increased delta band activity was normalized after 6 months of pharmacotherapy. Additionally, higher absolute theta activity at baseline predicted a greater possibility of improved addiction symptoms following treatment, but these neurophysiological changes were not related to depressive or anxiety symptoms because depressive or anxiety symptoms did not show significant changes following treatment; there were no correlations between the reduction of the frontal delta band activity and depressive or anxiety symptoms changes following treatment; and higher absolute theta activity in the central region at baseline predicted a greater possibility of improved addiction symptoms in IGD patients with comorbid issues following treatment, even though the effects of depressive or anxiety symptoms at baseline were controlled. The present findings indicated that slow-wave activity in IGD patients may be a state marker associated with changes in addiction symptoms following treatment.

Resting-state EEG activity represents the readiness or potential of the brain to allocate neural resources.^[27]^ The default mode network is a network of brain regions that exhibits increased activity during the resting state, and the intrinsic activities within these areas are known to impact subsequent stimulus or task-induced activity.^[28]^ Therefore, investigating resting-state brain activity may further the current understanding of basic brain functions. Delta and theta wave activities are involved in a variety of cognitive processes. For example, increased theta power is observed during tasks that demand attention, which suggests that low-frequency bands are involved in higher-order control processes.^[29]^ Moreover, event-related P300 responses are primarily the outcome of delta and theta oscillations elicited during the cognitive processing of stimuli.^[30,31]^ These P300-related oscillations are associated with different cognitive functions. For example, theta is associated with memory processes or attention and is considered to be an index of frontal processing, whereas delta is related to signal detection and decision-making and is generated by cortico–cortical interactions.^[32]^ Patients with AUD exhibit significantly lower evoked delta and theta oscillation amplitudes during the processing of target stimuli.^[33]^ Similarly, a recent study reported that the auditory P300 amplitude is reduced in patients with IGD compared with healthy controls.^[34]^ Given that resting-state brain activity impacts subsequent task-induced activity, it is of interest to note that increased delta and theta activities during the resting state may be related to decreased task-related P300 amplitudes in IGD patients. Additionally, EEG activity in the slow frequency bands may be associated with inhibitory control. For example, Schiller et al ^[35]^ found that increased power in the slow frequency bands of the lateral prefrontal cortex is linked to inhibitory deficits and, moreover, impairments in inhibitory function may occur in patients with IGD. Therefore, increased delta and theta activities during the resting state are likely associated with dysfunctional inhibitory control in IGD patients.

In the present study, IGD patients exhibited significant improvements in their addiction symptoms. Additionally, the increased delta band activity observed at baseline in this group was normalized after treatment and was significantly correlated with improvements in IGD symptoms. Moreover, the present study found that higher absolute theta activity at baseline predicted a greater possibility of improvement in IGD symptoms after treatment. After controlling for the effects of depressive or anxiety symptoms, these findings remained significant. There have been mixed results regarding changes in slow-wave activity and their associations with treatment in AUD patients. Saletu-Zyhlarz et al^[12]^ reported that abstaining patients showed an increase in slow activity after 6 months of treatment compared with baseline, and Pollock et al^[36]^ found persistent slow-wave alterations in alcoholics with prolonged abstinence. These authors also suggested that the higher power of the slow waves could be considered a trait marker of AUD.^[36]^ In contrast, the present study found that IGD patients displayed a decrease in absolute delta power; in other words, absolute delta power in this population normalized after 6 months of treatment and was associated with an improvement in IGD symptoms. This finding indicates that there may be different mechanisms underlying the neurophysiological activities associated with symptom changes in AUD and IGD patients. As mentioned above, slow-wave activities are involved in various types of cognitive processes;^[26–28]^ thus, the normalized delta power in IGD patients following treatment was likely related to changes in cognitive function. Accordingly, patients with Alzheimer disease exhibit significant decreases in power in the delta and theta bands during treatment with rivastigmine, which is an acetylcholinesterase inhibitor, indicating that there is a shift of the power spectrum toward normalization.^[37]^ In the future, it will be necessary to determine the relationships of the neurophysiological changes in IGD patients after treatment with changes in cognitive function. In addition, further methodological variations should be considered in an attempt to characterize electrical processes across the cortex, such as center of mass of EEG equation to visualize EEG traveling moves,^[38]^ and spatiotemporal analysis calculating the velocity direction and progresses in the 2-dimensional vector field of delays.^[39]^

The present study found that IGD patients with comorbid psychiatric issues had increased delta and theta powers compared with healthy controls. Previous studies observed increases in absolute delta and relative theta powers in IGD patients with comorbid depression compared with those with pure IGD, whereas patients with pure IGD show decreased absolute delta or beta powers.^[10,11]^ In addition, patients with depression exhibit increased slow-wave activities.^[40,41]^ Taken together, these findings indicate that comorbid psychiatric issues, which are frequently observed in patients with IGD, influence the brain function of this population.

The present study was subject to certain limitations. First, the sample size was too small to represent the overall characteristics of IGD patients, and only male subjects were included. Thus, the generalizability of the present results is limited. However, given that the point prevalence of IGD in 15 to 19 years-old was 8.4% for males and 4.5% for females,^[1]^ this apparent predisposition on the part of male participants suggests the basic differences in the way each sex utilizes the Internet activity. Second, the treatment modalities utilized in the present study were not well organized but consisted of usual outpatient care. However, the main focus of this study was on determining the neurophysiological markers associated with longitudinal symptom changes rather than the effects of the treatment itself. In the future, it will be necessary to investigate the effects of specific treatment modalities on the neurophysiological markers of IGD patients. Third, all participants with IGD in the present study had comorbid depressive or anxiety symptoms that may have influenced changes in EEG data. However, the effects of these depressive and anxiety symptoms were controlled for, and there were no differences in EEG activities between the IGD patients with depressive symptoms and those with anxiety symptoms.

Lastly, we carried out resting-state EEG spectral analysis based on the definition of normative frequency bands and recording references provided by internationally standardized 10 to 20 system. To investigate the specific activity of local brain sources, further analysis using current source density measures of EEG have to be taken into account.

In summary, the present findings demonstrated that there was an increase in resting-state slow-wave activity at baseline in IGD patients with comorbidities but that this increased delta band activity was normalized after 6 months of outpatient treatment. Furthermore, higher absolute theta activity at baseline predicted a greater possibility of improvements in addiction symptoms after treatment, even after adjusting for the effects of depressive or anxiety symptoms. These findings indicate that the increased slow-wave activity observed in the present study represents a state neurophysiological marker and that increased theta activity at baseline may be considered a favorable prognostic marker for IGD patients with comorbid depressive or anxiety disorders. Furthermore, it is important to note that most patients with IGD in clinical settings have various types of comorbid issues; thus, the present findings may provide useful clinical information regarding the neurobiological markers associated with IGD.

## Acknowledgments

The authors thank Jae-A Lim, Ji Yoon Lee, and Su Mi Park for helping to collect data.

## References

[1] Arlinton, VA, American Psychiatric Association Diagnostic and Statistical Manual of Mental Disorders: DSM-5. 5th ed. 2013.

[2] Ha JH Yoo HJ Cho IH Psychiatric comorbidity assessed in Korean children and adolescents who screen positive for Internet addiction. J Clin Psychiatry 2006;67:821–6. 16841632 10.4088/jcp.v67n0517

[3] Yen JY Ko CH Yen CF The comorbid psychiatric symptoms of Internet addiction: attention deficit and hyperactivity disorder (ADHD), depression, social phobia, and hostility. J Adolesc Health 2007;41:93–8. 17577539 10.1016/j.jadohealth.2007.02.002

[4] Bernardi S Pallanti S Internet addiction: a descriptive clinical study focusing on comorbidities and dissociative symptoms. Compr Psychiatry 2009;50:510–6. 19840588 10.1016/j.comppsych.2008.11.011

[5] Zhang L Amos C McDowell WC A comparative study of Internet addiction between the United States and China. Cyberpsychol Behav 2008;11:727–9. 18991530 10.1089/cpb.2008.0026

[6] Barry RJ Clarke AR Johnstone SJ EEG differences between eyes-closed and eyes-open resting conditions. Clin Neurophysiol 2007;118:2765–73. 17911042 10.1016/j.clinph.2007.07.028

[7] Porjesz B Begleiter H Alcoholism and human electrophysiology. Alcohol Res Health 2003;27:153–60. 15303626 PMC6668890

[8] Canuet L Ishii R Pascual-Marqui RD Resting-state EEG source localization and functional connectivity in schizophrenia-like psychosis of epilepsy. PLoS One 2011;6:e27863. 22125634 10.1371/journal.pone.0027863 PMC3220705

[9] Choi JS Park SM Lee J Resting-state beta and gamma activity in Internet addiction. Int J Psychophysiol 2013;89:328–33. 23770040 10.1016/j.ijpsycho.2013.06.007

[10] Son KL Choi JS Lee J Neurophysiological features of Internet gaming disorder and alcohol use disorder: a resting-state EEG study. Transl Psychiatry 2015;5:e628. 26327686 10.1038/tp.2015.124 PMC5068800

[11] Lee J Hwang JY Park SM Differential resting-state EEG patterns associated with comorbid depression in Internet addiction. Prog Neuropsychopharmacol Biol Psychiatry 2014;50:21–6. 24326197 10.1016/j.pnpbp.2013.11.016

[12] Saletu-Zyhlarz GM Arnold O Anderer P Differences in brain function between relapsing and abstaining alcohol-dependent patients, evaluated by EEG mapping. Alcohol Alcohol 2004;39:233–40. 15082461 10.1093/alcalc/agh041

[13] Wang GY Kydd R Wouldes TA Changes in resting EEG following methadone treatment in opiate addicts. Clin Neurophysiol 2014;126:943–50. 25301768 10.1016/j.clinph.2014.08.021

[14] Anderson NE Baldridge RM Stanford MS P3a amplitude predicts successful treatment program completion in substance-dependent individuals. Subst Use Misuse 2011;46:669–77. 21039117 10.3109/10826084.2010.528123

[15] Young KS Psychology of computer use: XL. Addictive use of the Internet: a case that breaks the stereotype. Psychol Rep 1996;79:899–902. 8969098 10.2466/pr0.1996.79.3.899

[16] Király O Griffiths MD Demetrovics Z Internet gaming disorder and the DSM-5: conceptualization, debates, and controversies. Curr Addict Rep 2015;2:254–62.

[17] Barke A Nyenhuis N Kröner-Herwig B The German version of the internet addiction test: a validation study. Cyberpsychol Behav 2012;15:534–42. 10.1089/cyber.2011.0616 23002984

[18] Fernández-Villa T Molina AJ García-Martín M Validation and psychometric analysis of the Internet Addiction Test in Spanish among college students. BMC Public Health 2015;15:1. 26400152 10.1186/s12889-015-2281-5 PMC4581075

[19] Widyanto L McMurran M The psychometric properties of the internet addiction test. Cyberpsychol Behav 2004;7:443–50. 15331031 10.1089/cpb.2004.7.443

[20] Lee K Lee H-K Gyeong H Reliability and validity of the Korean version of the Internet addiction test among college students. J Korean Med Sci 2013;28:763–8. 23678270 10.3346/jkms.2013.28.5.763 PMC3653091

[21] Beck AT Ward CH Mendelson M An inventory for measuring depression. Arch Gen Psychiat 1961;4:561–71. 13688369 10.1001/archpsyc.1961.01710120031004

[22] Beck AT Epstein N Brown G An inventory for measuring clinical anxiety: psychometric properties. J Consult Clin Psychol 1988;56:893–7. 3204199 10.1037//0022-006x.56.6.893

[23] Zeger SL Liang KY Longitudinal data analysis for discrete and continuous outcomes. Biometrics 1986;42:121–30. 3719049

[24] McLoughlin G Albrecht B Banaschewski T Electrophysiological evidence for abnormal preparatory states and inhibitory processing in adult ADHD. Behav Brain Funct 2010;6:66. 21029446 10.1186/1744-9081-6-66 PMC2988695

[25] Claassen J Hirsch LJ Kreiter KT Quantitative continuous EEG for detecting delayed cerebral ischemia in patients with poor-grade subarachnoid hemorrhage. Clin Neurophysiol 2004;115:2699–710. 15546778 10.1016/j.clinph.2004.06.017

[26] Wang X Sun J Gustafson KJ Modeling heterogeneity and dependence for analysis of neuronal data. Stat Med 2007;26:3927–45. 17577244 10.1002/sim.2943

[27] Thatcher RW North D Biver C EEG and intelligence: relations between EEG coherence, EEG phase delay and power. Clin Neurophysiol 2005;116:2129–41. 16043403 10.1016/j.clinph.2005.04.026

[28] Andrews-Hanna JR Reidler JS Huang C Evidence for the default network's role in spontaneous cognition. J Neurophysiol 2010;104:322–35. 20463201 10.1152/jn.00830.2009 PMC2904225

[29] Nigbur R Ivanova G Sturmer B Theta power as a marker for cognitive interference. Clin Neurophysiol 2011;122:2185–94. 21550845 10.1016/j.clinph.2011.03.030

[30] Basar E Basar-Eroglu C Karakas S Oscillatory brain theory: a new trend in neuroscience. IEEE Eng Med Biol Mag 1999;18:56–66. 10.1109/51.765190 10337564

[31] Karakas S Erzengin OU Basar E A new strategy involving multiple cognitive paradigms demonstrates that ERP components are determined by the superposition of oscillatory responses. Clin Neurophysiol 2000;111:1719–32. 11018485 10.1016/s1388-2457(00)00418-1

[32] Chen AC Rangaswamy M Porjesz B John I Nurnberger J Berrettini WH Endophenotypes in psychiatric genetics. Principles of Psychiatric Genetics. New York:Cambridge University Press; 2012 347–62.

[33] Jones KA Porjesz B Chorlian D S-transform time-frequency analysis of P300 reveals deficits in individuals diagnosed with alcoholism. Clin Neurophysiol 2006;117:2128–43. 16926113 10.1016/j.clinph.2006.02.028

[34] Park M Choi JS Park SM Dysfunctional information processing during an auditory event-related potential task in individuals with Internet gaming disorder. Transl psychiatry 2016;6:e721. 26812042 10.1038/tp.2015.215 PMC5068886

[35] Schiller D Kanen JW LeDoux JE Extinction during reconsolidation of threat memory diminishes prefrontal cortex involvement. Proc Natl Acad Sci 2013;110:20040–5. 24277809 10.1073/pnas.1320322110 PMC3864277

[36] Pollock VE Schneider LS Zemansky MF Topographic quantitative EEG amplitude in recovered alcoholics. Psychiatry Res 1992;45:25–32. 1410076 10.1016/0925-4927(92)90011-r

[37] Adler G Brassen S Chwalek K Prediction of treatment response to rivastigmine in Alzheimer's dementia. J Neurol Neurosurg Psychiatry 2004;75:292–4. 14742608 PMC1738912

[38] Manjarrez E Vázquez M Flores A Computing the center of mass for traveling alpha waves in the human brain. Brain Res 2007;1145:239–47. 17320825 10.1016/j.brainres.2007.01.114

[39] Massimini M Huber R Ferrarelli F The sleep slow oscillation as a traveling wave. J Neurosci 2004;24:6862–70. 15295020 10.1523/JNEUROSCI.1318-04.2004 PMC6729597

[40] Adler G Bramesfeld A Jajcevic A Mild cognitive impairment in old-age depression is associated with increased EEG slow-wave power. Neuropsychobiology 1999;40:218–22. 10559706 10.1159/000026623

[41] Morita A Kamei S Sakai T Relationship between quantitative electroencephalogram and interferon-alpha-induced depression in chronic hepatitis C patients. Neuropsychobiology 2013;67:122–6. 23406654 10.1159/000346091

